# Sociocultural factors in relation to mental health within the Inuit population of Nunavik

**DOI:** 10.17269/s41997-022-00705-w

**Published:** 2022-11-07

**Authors:** Natalia Poliakova, Mylene Riva, Christopher Fletcher, Mireille Desrochers-Couture, Yohann Courtemanche, Caroline Moisan, Sarah Fraser, Camille Pépin, Richard E. Bélanger, Gina Muckle

**Affiliations:** 1 Research Centre of CHU de Québec-Université Laval, Quebec City, Quebec Canada; 2 Institute for Health and Social Policy, McGill University, Montreal, Quebec Canada; 3 Department of Geography, McGill University, Montreal, Quebec Canada; 4 Department of Social and Preventive Medicine, Université Laval, Quebec City, Quebec Canada; 5 School of Psychology, Université Laval, Quebec City, Quebec Canada; 6 Faculty of Arts and Science, Université de Montréal, Montreal, Quebec Canada; 7 Faculty of Medicine, Université Laval, Quebec City, Quebec Canada

**Keywords:** Mental health, Depression, Suicide, Inuit, Social determinants of health, Santé mentale, dépression, suicide, Inuits, déterminants sociaux de la santé

## Abstract

**Objective:**

Built on the Inuit determinants approach of health, this study aimed to identify sociocultural factors associated with mental health among Inuit of Nunavik to guide programs and services.

**Methods:**

The data were collected through the *Qanuilirpitaa?* 2017, a survey characterized by the involvement of several Inuit representatives. Depressive symptoms (10-item Center for Epidemiologic Studies-Depression scale, CES-D), lifetime suicide ideation and attempts, and past-year ideation were self-reported mental health indicators. Sociocultural factors represented four thematic domains: social support, community activities, traditional practices, and cultural identity. Analyses tested whether the sociocultural factors were associated with indicators of mental health using weighted multivariate regressions.

**Results:**

Among the sociocultural factors considered, family cohesion and weekly hunting/fishing activities were associated with lower depression scores. Community cohesion and lower cultural identity (centrality scale) were associated with a lower likelihood of past-year and lifetime ideation while family cohesion was related to a lower likelihood of lifetime attempts. People with psychological distress (higher CES-D, suicidal ideation or attempts) were more likely to participate in healing and wellness activities.

**Conclusion:**

Although limited by their cross-sectional character, these analyses, based on the community component of the *Qanuilirpitaa?*, suggest that strengthening of family and community cohesion, and support of regular hunting and fishing deserve further attention as potential cumulative preventive avenues for Inuit mental health.

**Supplementary Information:**

The online version contains supplementary material available at 10.17269/s41997-022-00705-w.

## Résumé

**Objectif:**

Basée sur la conception Inuit des déterminants de la santé, cette étude identifie des facteurs socioculturels associés à la santé mentale chez les Inuit du Nunavik afin de guider les programmes et les services de santé.

**Méthode:**

Les données proviennent de *Qanuilirpitaa? 2017,* une enquête ayant impliqué plusieurs représentants Inuit. Les symptômes dépressifs (échelle *Center for Epidemiologic Studies-Depression*, 10 items, CES-D), les idéations suicidaires et les tentatives de suicide à vie, et les idéations au cours des derniers mois furent les indicateurs de santé mentale analysés. Des facteurs socioculturels investigués représentaient, eux, quatre domaines : support social, activités communautaires, pratiques traditionnelles et identité culturelle. Des régressions multivariées pondérées ont testé les associations entre les facteurs socioculturels et la santé mentale.

**Résultats:**

Parmi les facteurs socioculturels investigués, la cohésion familiale et une pratique hebdomadaire de chasse/pêche étaient associées au score dépressif inférieur. La cohésion communautaire et une identité culturelle (centralité) plus faible s’associaient à une probabilité moindre d’idéations suicidaires à vie et au cours des 12 derniers mois; la cohésion familiale était associée à une probabilité moindre des tentatives suicidaires à vie. Les personnes rapportant une détresse supérieure (CES-D élevé, idéations ou tentatives) participaient davantage à des activités de guérison/de bien-être.

**Conclusion:**

Limitées par leur caractère transversal, ces analyses, inspirées du volet communautaire de *Qanuilirpitaa?,* suggèrent qu’un renforcement de la cohésion familiale et communautaire, ainsi qu’un soutien d’activités de chasse et de pêche régulières méritent davantage d’attention en tant qu’avenues préventives cumulatives potentielles pour la santé mentale des Inuit.

## Introduction

Mental health is part of a broader concept of mental wellness, also including prevention of mental illness, violence, substance misuse, and suicide (Alianait Inuit-Specific Mental Wellness Task Group, 2007). Drawing on Inuit traditional knowledge, the Alianait Inuit Mental Wellness Task Group recognized mental health as a core component of wellness overall for Inuit communities. The Nunavik health survey conducted in 1992 and the 2004 Nunavik Inuit Health Survey (NIHS 2004) reported tremendously high rates of psychological distress (Kirmayer & Paul, 2007). The NIHS 2004 revealed that about 13% of the population had high levels of depressive and anxiety symptoms, 34% reported having had suicidal ideation in their lifetime, and 20% have ever attempted suicide. High rates were again reported in the 2017 Nunavik Inuit Health Survey *Qanuilirpitaa?* (NIHS 2017), where 39% of the Nunavimmiut were facing clinically significant depressive symptoms with unchanged rates of lifetime suicide attempts (23%) and a significant increase in lifetime suicidal ideation (41%; Muckle et al., 2020a). Women and youth were particularly at risk. Elsewhere, the rate of suicide completion was most prevalent among young Inuit men (Fraser et al., 2015; Kumar & Tjepkema, 2019).

Inuit perspectives on health and wellness are firmly rooted in the social determinants approach (Inuit Tapiriit Kanatami, 2014, 2016). As outlined in the community component of the NIHS 2017, an Inuit holistic conceptual model of health and well-being includes three key dimensions of health, i.e., bodily health (*Ilusirsusiarniq*), feeling of well-being (*Qanuinngisiarniq*), and harmonious relations among people sharing the same place (*Inuuqatigiitsianiq*) (Fletcher et al., 2021). These three dimensions describe health as grounded in cultural and social processes and fostered by the eight interconnected determinants: community, identity, family, food, land, knowledge, economy, and services.

In line with the Inuit sociocultural determinants approach of health, previous quantitative and qualitative studies described the relations between different sociocultural factors and mental health. Thus, strong, close-knit communities with supporting and trusted networks were identified by the Labrador Inuit youth as an important factor promoting mental health (Salusky et al., 2021). Likewise, the protective role of perceived emotional support was described (Gray et al., 2016). In addition, associations between mental wellness and different dimensions of cultural identity (e.g., centrality, cultural connectedness and belonging, cultural efficacy) have also been highlighted. For example, cultural connectedness, measured through identity, traditions, and spirituality subscales, was related to increased self-efficacy and sense of self for Canadian Indigenous youth (two Inuit participants; Snowshoe et al., 2017).

More recently, several bivariate associations between mental health and sociocultural factors have been reported using the data of the 2017 NIHS (Muckle et al., 2020a). However, simple prevalence estimates obtained for specific subgroups are of limited interest for prevention and treatment. This is especially true for observational cross-sectional studies when multiple interrelated factors can contribute to an outcome (Reichenheim & Coutinho, 2010). In this situation, if some conditions are respected (e.g., a steady population size over the study period, no selective survival from the outcome), multiple regression modeling can be an appropriate way to investigate potential etiological mechanisms by identifying specific, independent contributions of different exposure factors to an outcome (Reichenheim & Coutinho, 2010). With regard to sociocultural determinants of mental health, even if these factors belong to different social domains (e.g., social support provided either by family or community, self-healing activities, practice of traditional activities, or cultural identity), many of them are interrelated and specific associations of these factors with mental health among Inuit are unknown. This information is important as each of these factors could represent a potentially distinct opportunity to be integrated in prevention and promotion efforts. By consequence, this study’s objective was to identify sociocultural factors independently related to better mental health outcomes among Nunavimmiut, controlling for sociodemographic characteristics. In addition, considering sex inequalities in mental health outcomes, associations with sociocultural factors were investigated for the whole population and for men and women separately.

## Methods

### Sample

This study used the NIHS 2017 cross-sectional data from a non-proportional stratified sample of 1326 Inuit from Nunavik. This survey is a joint initiative of community representatives, regional government, public health organizations, and academic researchers, and was implemented to provide up-to-date information on mental and physical health as well as on the determinants of health among Nunavimmiut. Targeting the beneficiaries of the James Bay and Northern Quebec Agreement residing in Nunavik, aged 16 years and over, the NIHS 2017 was conducted in all 14 Inuit communities and gathered information using clinical and laboratory testing, medical records review, and computerized interviewer-administered questionnaires available in 3 languages (Inuktitut, English, and French).

In accordance with the NIHS 2017 Policy on the Management of Databases and Biological Samples and in recognition of the Ownership, Control, Access and Possession (OCAP®; First Nations Information Governance Centre, 2014) principles, the NIHS Data Management Committee (DMC), which includes community representatives and leaders, evaluated the usefulness of the study’s research questions for the region and approved the request for data access. The first draft of this manuscript was submitted to the DMC and the results were discussed in a co-interpretation approach taking into consideration Inuit culture and values. The final manuscript that reflects the comments provided by DMC members and a one-page plain language summary were sent to the DMC for knowledge transfer and dissemination purposes.

Informed consent was obtained from all individual participants included in the study. All survey materials, including consent form, were available in Inuktitut and interpreters were available at all times. The NIHS 2017 received ethical approval (#2016-2499-21) by the Research Ethics Board of CHU de Québec-Université Laval. Detailed information on the NIHS 2017 is provided in the Methodological Report (Hamel et al., 2020).

### Measures

To ensure tailoring of the questionnaire to Inuit culture, the selection of specific scales/questions has been done in collaboration with Inuit representatives where, among published and validated scales (whenever possible), those the most adapted to Inuit culture have been retained. Also, some standardized tools had to be adapted to represent the specificities of Nunavimmiut.

#### Mental health

Four self-reported indicators of mental health were used: past-week depressive symptoms, lifetime suicidal ideation and attempts, and suicidal ideation during the year prior to the survey. Depressive symptoms were documented using the 10-item version of the Center for Epidemiologic Studies-Depression rating scale (CES-D; Andresen et al., 1994). Validity and reliability of the CES-D have been established for a variety of populations (James et al., 2020), including those of Indigenous ancestry (Armenta et al., 2014). Participants were asked to rate the frequency of feelings (e.g., ‘*I felt depressed*’, ‘*I felt fearful*’, and ‘*I was happy*’) experienced during the week prior to the interview on a 4-point scale ranging from *1-All of the time* to *4-Rarely or none of the time*. One of the positive items, ‘*I felt hopeful about the future*’, presented low inter-item correlations and negatively affected the measure of internal consistency and, consequently, was omitted. Thus, the CES-D total score was calculated by summing the responses across 9 items after reversing the scale for negative items, with higher scores reflecting more severe depressive symptoms (9-item *α*_Cronbach_ = 0.78). The 9-item CES-D scores were highly correlated to the original 10-item scores (*r* = 0.98, *p*<0.0001). Dichotomized CES-D scores were also generated with a cut-off point of 9 or higher, which is an equivalent of the official clinical cutting point of 10 on the 10-item CES-D. Three Yes/No questions assessed suicidal ideation and attempts: (1) ‘*Have you ever thought seriously about committing suicide (taking your life)?*’; (2) ‘*Have you ever attempted suicide (tried to take your life)?*’; (3) ‘*In the past 12 months, have you thought seriously about committing suicide?*’.

#### Sociocultural factors

We retained several sociocultural factors highlighted previously as relevant to Inuit mental health and wellness (e.g., Gray et al., 2016; Muckle et al., 2020a) and those identified through the consultation process with the community representatives. As described in Table 1, these factors include three measures of perceived social support and within-group cohesion; a set of behaviours and value perceptions related to traditional activities and practices; participation in different activities that represent opportunities to strengthen social ties and to receive social support; and, finally, two dimensions of cultural identity (i.e., centrality and connectedness). Centrality refers to the extent to which individuals feel their native culture as a central or important part of the self (Schwartz et al., 2014); connectedness (Snowshoe et al., 2017) describes perception of connections to Inuit elders, youth, and other aboriginal people, and feeling comfortable with Inuit and non-Inuit. All sociocultural factors were grouped into four thematic blocks: (1) social support, (2) community activities, (3) traditional activities, and (4) cultural identity.

**Table 1:** Description of sociocultural indicators

Indicator	Description
Block 1. Social support	
Affective social support	5 questions on frequency of three types of social support: (Richmond et al., 2007) - Positive interactions: “Have someone to have a good time with” - Emotional support: “Have someone to talk if I feel troubled or need emotional support”, “Have someone to count on when I need advice”, “Have someone to listen when I need to talk” - Love and affection: “Have someone who shows me love and affection” Likert scale (1-All of the time to 5-Never) Continuous score calculated by summing reversed scale (Cronbach *α* = 0.79).
Family cohesion	6 questions: 5 from the Brief Family Relationship Scale (Fok et al., 2014) + one item to adapt to Inuit culture. “In my close family… “there is a feeling of togetherness”, “we really help and support each other”, “we really get along well with each other”, “we spend a lot of time doing things together at home”, “we spend a lot of time doing things together on the land”, “I am proud to be part of my family”” Likert scale: 1-Very true to 3-Not true Continuous score calculated by summing reversed scale (Cronbach *α* = 0.78).
Community cohesion	4 questions on perception of social cohesion in the community: “There is a feeling of togetherness or closeness”, “People help each other”, “People can be trusted”, “I feel like I belong” Likert scale: 1-Strongly agree to 5-Strongly disagree Continuous score calculated by summing reversed scale (Cronbach *α* = 0.78).
Block 2. Traditional activities	
Frequency of going on the land	“From the Spring until now, how often did you go on the land?” Likert scale: 1-Never, 2-Occasionally, 3-Often Comparisons: 1-Often vs.0-Never or Occasionally.
Hunting and fishing	The highest frequency of hunting and fishing on four seasons during the previous 12 months Likert scale: 1-Never, 2-Less than once a month, 3-1 to 3 days per month, 4-Once a week or more Comparisons: 1-Hunting or fishing at least weekly vs. 0-Hunting or fishing less than weekly or never.
Harvesting seafood	The highest frequency of harvesting seaweeds, mollusks, and urchins on four seasons in the previous 12 months Likert scale: 1-Never, 2-Less than once a month, 3-1 to 3 days per month, 4-Once a week or more Comparisons: 1-Harvesting at least monthly vs. 0-Harvesting less than monthly or never.
Berry picking	Frequency of seasonal berry picking in the previous 12 months Likert scale: 1-Never, 2-Less than once a month, 3-1 to 3 days per month, 4-Once a week or more Comparisons: 1-Berry picking practiced at least monthly vs. 0-Berry picking practiced less than once a month or never.
Abilities to practice traditional activities	4 questions. How satisfied are you with your… “ability to go on the land hunting, fishing or berry picking”, “ability to satisfy country food cravings”, “ability to communicate with others in Inuktitut”, “knowledge and skills of cultural and traditional activities, games, and arts” Likert scale: 1-Very satisfied to 5-Very dissatisfied Continuous score calculated by adding reversed scale (Cronbach *α* = 0.64).
Importance of spiritual values	“Do spiritual values play an important role in your life?” Yes/No answer
Block 3. Community activities	
Participation in religious activities	“During the past 12 months, not counting events such as weddings or funerals, how often did you participate in religious activities or attend religious services or meetings?” Likert scale: 1-Never to 4-One or few times a week Comparisons: 1-participation at least once a month vs.0-less than once a month.
Involvement in community activities	Frequency of involvement in two types of community activities: “Participation in cultural community or sporting events such as festivals, dances, feasts or Inuit games”, “Volunteered for a group, organization, community event, rescue team, church group, feasts, spring clean-up” Likert scale: 1-Always to 5-Never Continuous score calculated by summing reversed scale (Cronbach *α* = 0.63).
Participation in healing and wellness activities	“In the past 12 months, have you taken part in any activities to promote your own healing or wellness?” Yes/No answer
Block 4. Cultural identity	
Centrality	5 questions on importance of Inuit values from the Pacific Identity and Wellbeing Scale (Manuela & Sibley, 2013), adapted to Inuit culture: “…Inuit is an important part of my identity”, “Going on the land is an important part of my life”, “…sharing is an important Inuit value”, “… Inuktitut is an important part of my identity”, “…proud to be Inuk” Likert scale: 1-Strongly agree to 5-Strongly disagree Continuous score calculated by adding reversed scale (Cronbach *α* = 0.76).
Connectedness	6 questions on feeling of connection to Inuit and non-Inuit from the Pacific Identity and Wellbeing Scale (Manuela & Sibley, 2013), adapted to Inuit culture: “…feel comfortable around other Inuit”, “…feel comfortable with non-Inuit”, “…feel connected to other aboriginal people”, “…close connections to elders”, “…close connections to youths”, “...like travelling outside of Nunavik” Likert scale: 1-Strongly agree to 5-Strongly disagree Continuous score calculated by adding reversed scale (Cronbach α = 0.65).

#### Sociodemographic characteristics

Age, sex (men vs. women), marital status (single vs. married or common law vs. separated, divorced or widowed), highest education level (from 1 = Grade 1 to 15 = Graduated from university), employment (employed vs. not employed), and annual personal income (less than $20,000 vs. $20,000 and over) were self-reported. Coastal region of residence (Hudson vs. Ungava) and community size (small vs. large) were also considered.

### Analyses

Statistical analyses were conducted using SAS 9.4 (SAS Institute Inc., Cary, NC, United States) for the whole population and for men and women separately. Bootstrap weights were included in the analyses to ensure the representativeness of the Nunavik population. First, the distribution of sociodemographic characteristics and the prevalence of mental health outcomes were examined using chi-square and variance estimation analyses. Second, regression models, one per block of sociocultural factors, were used to examine the associations with mental health indicators and to select those to be included in the next step. To describe specific associations with sociocultural factors from different blocks, between-block regression analyses included all sociocultural factors associated with mental health outcomes with *p* < 0.10 at the step of within-bloc analyses. With regard to the CES-D, analyses using both continuous and dichotomized scores were performed. The first one avoids loss of information while the second facilitates interpretation and allows comparisons with previous studies having used the clinical cut-off. In all regression analyses, the above-mentioned sociodemographic characteristics were considered potential confounding factors. As inclusion of annual income, region of residence and community size did not change any regression coefficient in either direction by 10% or more, these variables were not considered in the final regression models. Also, as suggested through the review process, we have also considered inclusion of participation in healing and wellness practices as potential confounder in the within-block analyses. Inclusion of this variable did not change the results. By consequence, the within-block results without this supplemental variable are reported. Correlations between sociocultural factors and mental health outcomes, and intercorrelations between sociocultural factors for men and women were also performed and results are available as supplemental materials (Table S1). Statistical significance was set at *p* < 0.05.

## Results

### Description of sociodemographic characteristics and mental health

Weighted descriptive results for mental health, sociocultural, and sociodemographic indicators are reported in Table 2. In 2017, there were equal proportions of men (50.4%) and women among Nunavimmiut aged 16 years and older with nearly half residing on the Hudson coast (56.6%) versus the Ungava coast. About half (52.2%) of Nunavimmiut were in a common-law relationship or married; 60.5% of people had incomplete secondary (high school) education and 68.3% were in the paid labour force. Mean CES-D score was 7.6 and 37% of people were identified as having clinically significant depressive symptoms (cut-off score of 9), with a higher prevalence among women than among men (42% versus 32%). Lifetime suicidal ideation and attempts, and past-year ideation were reported by 41.0%, 29.8%, and 14.9% of people, respectively. Higher CES-D score and higher prevalence of suicidal ideation and attempts were observed for women. Also, significantly higher CES-D scores were observed both for men and women having reported suicide ideation or attempts (data not shown).

**Table 2:** Sociodemographic characteristics and sociocultural and mental health indicators with comparisons between sexes

	Total		Men		Women		
	%	*Mean*	%	*Mean*	%	*Mean*	*p*^*1*^
Sociodemographic characteristics							
Sex			50.39		49.61		NA
Age (years)							
16–30	43.86		43.87		43.85		0.34
31–54	39.33		38.59		40.09		
55 and over	16.81		17.54		16.06		
Marital status							
Single	42.20		42.53		41.87		0.04
Married or common law	52.24		53.60		50.86		
Separated, widowed, or divorced	5.56		3.87		7.26		
Education (highest grade completed)							0.04
Elementary school or less	10.15		12.34		7.90		
Secondary school not completed	60.48		60.40		60.57		
Secondary school or higher	29.37		27.26		31.53		
Employment (% working)	68.25		69.40		67.09		0.41
Annual personal income							0.33
<$20K	46.85		48.40		45.11		
$20K or more	53.15		51.60		54.89		
Coast of residence							NA
Hudson Coast	56.63		56.97		56.27		
Ungava Coast	43.37		43.03		43.73		
Community size							0.04
Small	42.66		43.39		41.91		
Large	57.34		56.61		58.09		
Sociocultural characteristics							
Block 1. Social support							
Affective social support		13.30		12.74		13.87	<0.001
Family cohesion		7.12		7.27		6.97	0.02
Community cohesion		11.56		11.95		11.17	<0.001
Block 2. Traditional practices							
Going on the land (often)	43.56		44.86		42.25		0.39
Hunting/Fishing (at least weekly)	62.60		68.86		56.29		<0.001
Harvesting (at least monthly)	35.41		38.21		32.59		0.03
Berry picking (at least monthly)	52.82		38.08		67.81		<0.001
Ability to practice traditional activities		16.97		17.02		16.91	0.43
Importance of spiritual values, yes	82.63		80.32		85.00		0.05
Block 3. Participation in community activities							
Religious activities (at least monthly)	40.7		36.20		45.35		0.002
Volunteering and community activities		4.05		4.22		3.88	0.01
Healing and wellness activities, yes	30.16		27.13		33.27		0.03
Block 4. Cultural identity							
Centrality		23.02		22.93		23.10	0.23
Connectedness		24.51		24.67		24.34	0.05
Mental health indicators							
CES-D, continuous		7.60		7.21		7.99	0.01
CES-D, higher symptoms	36.97		32.22		41.79		0.002
Lifetime							
Suicide ideation, yes	41.01		35.15		47.00		<0.001
Suicide attempts, yes	29.78		26.26		33.38		0.02
Past 12 months							
Suicide ideation, yes	14.92		12.58*		17.31		0.03

*NA*: non-available because the variable is used to create weights and the variance estimate of the test statistic is zero. ^1^*p* values of comparisons between sexes

*Coefficient of variation greater than 15% and lower than or equal to 25%. Proportion should be interpreted carefully

Note*: t*-tests were performed for continuous and Wald log-linear *χ*^2^ test for categorical variables

### Associations of sociocultural factors with mental health indicators

As sex-specific associations between sociocultural factors and mental health outcomes were very similar for men and women, results by sex are available as supplemental materials (Tables S2 and S3) while results for the whole population are reported and discussed in the text.

#### Depression

The within-block analyses (see Table 3) revealed that higher family cohesion and frequent participation in hunting and fishing activities were significantly related to lower CES-D scores. Thus, using the CES-D dichotomized score, the odds of getting into the low CES-D group was 12% (1/AOR*100%) higher with each unit increase on the family cohesion scale and 84% higher for people participating at least weekly in hunting/fishing activities. Participation in activities promoting health and wellness was more likely to be seen in those with higher CES-D scores, while cultural identity variables were not related to depression scores.

**Table 3:** Within-block multivariate regression analyses for mental health indicators by sociocultural factors

	CES-D		12-mo ideation		Lifetime ideation		Lifetime attempts	
	Std *ß*	AORs^1^ [95% CI]	AORs^1^ [95% CI]					
Block 1. Social support								
Affective social support	0.01	1.00 [0.96–1.04]	1.00	[0.94–1.06]	1.03	[0.98–1.07]	1.03	[0.99–1.07]
Family cohesion	−0.16*	0.89* [0.83–0.95]	0.95	[0.86–1.05]	0.96	[0.89–1.03]	**0.94**^**Ϯ**^	[0.87–1.01]
Community cohesion	0.01	1.03 [0.98–1.09]	0.93*	[0.86–0.99]	0.91*	[0.86–0.96]	0.95	[0.90–1.01]
Block 2. Traditional practices								
Going on the land (ref=occasionally or never)	0.02	1.09 [0.80–1.50]	0.95	[0.62–1.47]	0.90	[0.67–1.20]	1.04	[0.76–1.43]
Hunting/Fishing (ref=less than weekly)	−0.15*	0.54* [0.40–0.74]	0.74	[0.47–1.17]	1.12	[0.80–1.56]	0.76	[0.54–1.07]
Harvesting (ref=less than monthly)	0.05	0.27 [0.93–1.73]	1.16	[0.77–1.74]	0.88	[0.66–1.18]	1.00	[0.72–1.38]
Berry picking (ref=less than monthly)	−0.01	0.99 [0.73–1.36]	0.90	[0.57–1.41]	1.06	[0.78–1.44]	0.88	[0.64–1.22]
Ability to practice traditional activities	**−0.06**^**Ϯ**^	**0.94**^**Ϯ**^ **[0.88–1.00]**	1.02	[0.94–1.10]	0.98	[0.93–1.04]	1.04	[0.97–1.11]
Importance of spiritual values (ref=no)	0.03	1.20 [0.84–1.73]	0.73	[0.44–1.22]	0.79	[0.54–1.15]	1.23	[0.81–1.86]
Block 3. Participation in community activities								
Religious activities (ref=less than monthly)	−0.04	0.82 [0.62–1.08]	0.94	[0.61–1.43]	0.90	[0.67–1.21]	1.14	[0.84–1.55]
Volunteering and community activities	−0.02	1.00 [0.93–1.06]	1.01	[0.92–1.10]	**0.96**^**Ϯ**^	**[0.90–1.02]**	0.95	[0.89–1.02]
Healing and wellness activities (ref=no)	0.09*	1.55* [1.14–2.11]	1.53*	[1.02–2.28]	1.61*	[1.20–2.16]	1.67*	[1.22–2.29]
Block 4. Cultural identity								
Centrality	0.03	1.04 [0.97–1.11]	**1.09**^**Ϯ**^	**[0.99–1.19]**	**1.07**^**Ϯ**^	**[0.99–1.16]**	**1.08**^**Ϯ**^	**[0.99–1.17]**
Connectedness	−0.03	0.97 [0.93–1.02]	0.98	[0.91–1.06]	1.01	[0.96–1.06]	0.96	[0.91–1.02]

*AORs* adjusted odds ratios; *CI* confidence interval; *Std* standard

^1^CES-D total score was dichotomized at ≥ 9, which is also equivalent to the 66th percentile

Note: All analyses were adjusted for sex, age, marital status, education, and employment. Boldface indicates the variables included in the between-block analyses

*p < 0.05. ^Ϯ^*p* < 0.10

When all indicators at *p* < 0.10 were considered in between-block regression analyses (Table 4), results remained the same as significant associations were observed between higher family cohesion and at least weekly hunting and fishing activities, and lower CES-D, used either as continuous or dichotomized scores. Finally, the likelihood of participation in health and wellness activities was higher for people with higher CES-D scores.

**Table 4:** Between-block multivariate regression analyses for mental health indicators by sociocultural factors

	CES-D		12-month ideation		Lifetime ideation		Lifetime attempts	
	Std *ß*	AORs^1^ [95% CI]	AORs^1^ [95% CI]					
Block 1. Social support								
Family cohesion	**−0.13**	**0.92** [0.87–0.99]	**-**		**-**		**0.91**	[0.85–0.97]
Community cohesion	-		**0.89**	[0.83–0.96]	**0.90**	[0.85–0.96]		
Block 2. Traditional practices								
Hunting/Fishing (ref=less than weekly)	**−0.12**	**0.61** [0.46–0.81]	-		-		-	
Ability to practice traditional activities	−0.02	0.97 [0.91–1.03]	-		-		-	
Block 3. Participation in community activities								
Volunteering and community activities	**-**				0.97	[0.91–1.03]		
Healing and wellness activities (ref=no)	**0.11**	**1.72** [1.26–2.33]	**1.50**	[1.01–2.24]	**1.56**	[1.16–2.11]	**1.69**	[1.23–2.32]
Block 4. Cultural identity								
Centrality	-		**1.10**	[1.01–1.20]	**1.11**	[1.03–1.19]	1.06	[0.98–1.13]

*AORs* adjusted odds ratios, *CI* confidence interval, *Std* standard

^1^CES-D total score was dichotomized at ≥ 9, which is also equivalent to the 66th percentile

Note: All analyses were adjusted for sex, age, marital status, education, and employment. Boldface indicates significant estimates at *p* < 0.05

#### Suicide

Results from Table 3 indicated that perception of higher community cohesion was associated with a lower probability of both past-12-month and lifetime ideation, corresponding respectively to 8% and 10% lower likelihood of reporting the outcome with each unit increase on the community cohesion scale. Regarding self-help behaviours, participation in activities promoting health and wellness was more likely to be seen in people reporting past-12-month and lifetime ideation, and lifetime attempts.

These associations remained significant in the between-block analyses (Table 4). Also, significant associations were obtained for family cohesion and centrality of cultural identity. Thus, higher community cohesion was associated with a lower likelihood of both past-12-month and lifetime ideation while higher family cohesion was related to a lower likelihood of lifetime attempts. In addition, the odds of participation in health and wellness activities was higher for people experiencing past-12-month and lifetime suicide ideation, and lifetime attempts. Finally, higher centrality was related to a higher likelihood of past-12-month and lifetime ideation.

## Discussion

Using multivariate regressions, the objective of this study was to identify the sociocultural factors independently related to better mental health outcomes among Nunavimmiut, each of which could be a potential means of prevention and promotion of mental health and wellness. The analyses revealed significant specific positive associations with factors related to social support (i.e., family and community cohesion) and practice of traditional activities (i.e., weekly hunting/fishing). However, centrality of cultural identity and participation in healing and wellness activities were less frequently reported by people with better mental health.

As expected, perceived family cohesion was associated with lower depressive symptoms and a lower probability of lifetime suicide attempts, while perceived community cohesion was related to a lower likelihood of suicidal ideation. Regardless of cultural background, families with supportive and caring environment are frequently an important lasting resource for people struggling with mental health issues and have been suggested as a powerful determinant of mental health (Marsh & Johnson, 1997). In Inuit culture, family-based groups and communities have been historically forming fundamental social units (Inuit Tapiriit Kanatami, 2014). Over the last century, Inuit traditional family and community foundations have been challenged by settlements, overcrowding, substance use problems, and historical traumas (Petrasek MacDonald et al., 2015). Even if family and community relationships have been negatively affected by these factors, our results suggest that family and community continue to play an important role as protective factors for individuals’ mental health. Previous qualitative studies also highlighted family (e.g., Kral et al., 2011) or cohesive communities (e.g., Mohatt et al., 2004; Wexler & Goodwin, 2006) as most significant determinants of wellness for Inuit, while other studies identified community-level factors affecting family cohesion (e.g., family member physical health, housing conditions, formal services, and alcohol use in communities, as described in Fraser et al., 2018). As it was highlighted by consulted Inuit representatives, our results empirically support family- and community-based preventive and health promotion initiatives. Some of such programs are underway in Nunavik with the objective to better meet the needs of Nunavimmiut and their families. The Inuit representatives mentioned, for example, the ***Ilagiilluta*** **(Let’s be family)** Integrated Services in Perinatality and Early Childhood program, the development of the family houses network, and an inpatient family program to be provided by Isuarsivik Regional Recovery Centre.

Taking into consideration limited professional health services, Inuit families and communities may experience considerable difficulties and burden that emphasize the necessity of community- and family-oriented supportive and empowerment interventions and programs. Also, our family and community cohesion scales had both the items on help provided by other family and community members highlighting major contributions, among others, of natural helpers and family caregivers to health and well-being. In small communities, with relatively prevalent mental health problems, increased attention should also be brought to support these caregivers. Being a community member and an integral part of health services at the same time, natural helpers could be called upon to intervene with their relatives or have to deal with difficult personal situations with risk of experiencing powerlessness and a significant burden (Fraser et al., 2019; Lessard et al., 2008). National guidelines were developed to describe the needs of caregivers of family members living with mental health issues and to identify ways to effectively support caregivers and to mitigate their challenges (MacCourt et al., 2013). Future qualitative investigations could help to better define the specific needs of Inuit communities and to build culturally adapted supportive services.

In this study, people with higher depressive symptoms or reporting suicidal ideation or attempts were more likely to participate in activities promoting health and wellness. Even if no conclusion with regard to the direction of the observed associations nor causal connections can be drawn due to the cross-sectional design of the study, these latest results suggest that healing and wellness activities are reaching out to adults in need of support due to their distress. Mental health services available to Nunavimmiut appear to have improved since the beginning of the 2000s (Lessard et al., 2008) and are generally well known by the population (Muckle et al., 2020b). Nevertheless, only half of Nunavimmiut perceived services available in their communities as sensitive to Inuit reality (Muckle et al., 2020b).

Participation in traditional harvesting practices has been suggested as one of the key protective factors related to Inuit mental health and wellness (Inuit Tapiriit Kanatami, 2014). In this study, frequent involvement in hunting and fishing activities was related to lower depressive symptoms both for men and women (see Tables S1 and S2, Supplemental Materials). These land-based traditional activities often occur concurrently with socially bonding activities of sharing country food with family and community members. Both for men and women, participation in hunting and fishing activities presented specific associations with better mental health outcomes, over and above the associations with factors related to social support, such as perceived family and community cohesion. Different non-exclusive hypotheses could be mentioned to explain the associations. For men, beneficial psychosocial effects of hunting-fishing practices could be related to recovery of traditional roles and values of subsistence activities (Inuit Tapiriit Kanatami, 2014). For women, with about half of them having reported at least weekly participation in hunting/fishing activities, these practices were also associated with better mental health outcomes. On the land, women continue to hunt alongside men or by themselves; to fish; to collect berries, seaweeds, or eggs; and to prepare traditional food. These activities are ways to reconnect to Inuit culture, to enhance self-esteem and to foster supporting interpersonal relationships (Pauktuutit Inuit Women of Canada, 2006). For men and for women, the on-land activities also represent an important source of food (Pauktuutit Inuit Women of Canada, 2006). However, these activities are currently negatively impacted by climate change that reduces access to wildlife and threatens the safety of harvesters (Inuit Tapiriit Kanatami, 2014). Also, regardless of sex, the associations could be indirect through enhanced household food security, another known protective factor for Inuit mental health (Bradette-Laplante et al., 2020). In addition, the relation could be explained by high nutritious values of traditional food that have the potential to contribute to mental health (e.g., polyunsaturated fatty acids; Lucas et al., 2010). Finally, beneficial effects of eating traditional food providing a spiritual connection to the land and to Inuit traditional practices, as well as bonding effects of these activities (e.g., allowing young Inuit to spend more time with older ones and to learn traditional practices), were highlighted (Kral et al., 2011). Some examples of Inuit land-based mental wellness and prevention programs could be cited, such as *Aullak*, *sangilivallianginnatuk* (Going off, Growing Strong; Nain, Nunatsiavut), or, as mentioned by our Inuit collaborators, the on-land *Nunami* program developed by the Nunavik Regional Board of Health and Social Services to promote Nunavimmiut mental wellness.

With regard to cultural identity, higher centrality was independently associated with a higher likelihood of lifetime and past-12-month suicide ideation for Nunavimmiut and, more specifically, for women. Largely used in cultural and social psychology, centrality has been previously shown to be relevant in studying well-being within minority groups facing adversity, such as racial discrimination or identity-related stress. These previous studies have reported mixed results. For example, higher levels of racial centrality have been related to lower levels of subsequent psychological distress in a sample of African American students (Sellers et al., 2003) but also have demonstrated null relations with self-esteem within Latinos (Eccleston & Major, 2006). Elsewhere, higher centrality was associated with higher depressive (CES-D) scores at the bivariate level but, once combined with other identity subscales into ethnic identity profiles, those characterized by higher centrality in combination with higher scores on public and private regards and nationalism subscales were related to lower depressive scores (Yip et al., 2006). Two possible explanations for associations between higher centrality and higher psychological distress may be suggested. First, as an assumption made by our Inuit collaborators, the association can be mediated by other factors such as perceived discrimination. In that sense, individuals for whom their cultural identity is more central to the self may experience more frequently discriminatory events themselves from their cultural expression and, by consequence, higher psychological distress. Second, these individuals could also be more affected by collective cultural traumas or stigma, feeling more concerned by their group’s historico-cultural heritage. Research on the relation between centrality and mental health within Indigenous populations is very scarce. Results by Gfellner revealed no association between centrality and a scale of emotional tone in a sample of North American Indian/First Nation adolescents (Gfellner, 2016). In the study of Bombay et al. (2010), higher centrality was associated with higher depressive symptoms among First Nations adults, even after controlling for perceived discrimination. Also, the interaction between centrality and perceived discrimination was marginally significant (*p* = 0.07) in relation to the mental health outcome, with high centrality being related to depressive symptoms especially at higher levels of perceived discrimination, suggesting a more complex relation of centrality with mental health. All things considered, without further in-depth systematic investigation of the conditions when cultural centrality can be either a protective or risk factor for mental health, we can only speculate about its role. Also, cultural identity being a multi-dimensional and dynamic construct, interactions with other dimensions not examined in this study (e.g., dimensions of regard, salience, and ideology; Schwartz et al., 2014) in context with developmental processes should be considered to fully understand the relation between mental health and centrality, and, more globally, cultural identity.

Many significant sex differences for nearly all sociocultural determinants were observed. Also, in accordance with earlier studies within Arctic Indigenous people (e.g., Ragnhild Broderstad et al., 2011), higher depressive symptoms and higher rates of suicide ideation and attempts were reported by women, while the rates of suicide completion are known to be higher among Inuit men (Fraser et al., 2015; Kumar & Tjepkema, 2019), highlighting important sex inequalities in mental health. However, our results revealed quite similar associations between sociocultural determinants and mental health outcomes for both sexes (see Table S2, Supplemental Materials). This suggests that other individual, social, and cultural factors (e.g., teenage pregnancy and health care access for Inuit women, and chronic conditions and food security for Inuit men; Anderson, 2015; Healey & Meadows, 2008) might contribute in a differential way to observed sex disparities. Despite an increased acknowledgement of sex differences in health, there is a flagrant lack of research with sex-specific analyses among Indigenous populations (Nelson & Wilson, 2017).

The results of this study should be considered in light of certain limitations. With the goal to address knowledge gaps on culturally relevant determinants of mental health among Nunavimmiut, this study focused specifically on protective factors related to social support, family relations, culture, traditions, and life in community. Known risk factors such as victimization, physical and sexual abuse, childhood adverse events, substance use, and food insecurity also need to be considered to acquire a thorough understanding of determinants of mental health. In addition, the cross-sectional design of this study does not enable to conclude on causal effect of sociocultural factors. Indeed, even if many of structuring conditions required for testing causal relationships in cross-sectional studies (Reichenheim & Coutinho, 2010) were respected, the possibility of reverse causality could not be completely ruled out. By consequence, causal pathway hypothesized here should be addressed within a longitudinal design. Finally, the mental health indicators used in this study considered different timeframes: from the past week for CES-D to lifetime period for suicide ideation and attempts. It is possible that some mental health events could be prior to the timeframe targeted by some sociocultural determinants. However, the consistency of the results is reassuring and suggests timeless, steady nature of associations between mental health indicators and sociocultural determinants.

## Conclusion

This study revealed significant specific associations of family and community cohesion, frequent hunting and fishing, participation in healing activities, and centrality of cultural identity with mental health indicators among Nunavimmiut. Even if additional studies are needed to confirm the hypothesized causal relations, these results suggest that strengthening of family and community cohesion, and support of regular harvesting activities deserve further attention as promising avenues for mental health promotion programs and family- and community-oriented integrated strategies.

## Contributions to knowledge

What does this study add to existing knowledge?

- Using data from a large representative sample of the 2017 Nunavik Inuit Health Survey, this study identifies a number of major socio- and culturally relevant factors related to mental health among Nunavimmiut.
- Family and community cohesion and frequent participation in on-land activities such as hunting and fishing presented independent relations with better mental health outcomes.

What are the key implications for public health interventions, practice, or policy?

- The results provide empirical support to family- and community-oriented prevention and healing practices, activities, and programs.
- The results advocate for inclusion of traditional Inuit practices such as hunting and fishing in strategies designed to foster mental health.

### Supplementary information

ESM 1 (DOCX 28 kb)

## Data Availability

Inquiries regarding access to the data should be addressed to the survey’s Data Management Committee.
