# Supplementary material for: Sociocultural factors in relation to mental health within the Inuit population of Nunavik
Source: Can J Public Health. 2022 Nov 7;115(Suppl 1):83–95. doi: 10.17269/s41997-022-00705-w (PMC10830993; doi:10.17269/s41997-022-00705-w)
Supplement: Supplementary file 1 — (DOCX 28 kb) [file 41997_2022_705_MOESM1_ESM.docx]

**Supplemental material**

**Table S1**

*Intercorrelations for sociocultural factors and associations with mental health indicators for men (above the diagonal) and women (below the diagonal)*

| Factor | 1 | 2 | 3 | 4 | 5 | 6 | 7 | 8 | 9 | 10 | 11 | 12 | 13 | 14 | 15 | 16 | 17 | 18 |
| --- | --- | --- | --- | --- | --- | --- | --- | --- | --- | --- | --- | --- | --- | --- | --- | --- | --- | --- |
| 1. CES-D | - | **.20** | **.14** | **.18** | .01 | **-.21** | -.04 | -.09 | **-.15** | -.09 | -.09 | **-.15** | .01 | -.06 | **-.12** | **.13** | **-.12** | **-.12** |
| 1. Suicide ideation, lifetime (ref=No) | **.18** | - | **.42** | **.44** | .01 | **-.13** | **-.14** | -.04 | -.03 | -.07 | -.02 | -.06 | **-.14** | -.09 | -.07 | **.12** | -.04 | .00 |
| 1. Suicide attempts, lifetime (ref=No) | **.22** | **.49** | - | **.23** | .07 | **-.13** | -.01 | .01 | -.04 | -.07 | -.05 | .02 | -.02 | -.03 | -.08 | .07 | -.04 | -.08 |
| 1. Suicide ideation, past-12-mos (ref=No) | **.30** | **.34** | **.32** | - | .00 | **-.19** | **-.15** | -.04 | -.03 | -.02 | -.08 | -.09 | **-.16** | -.02 | -.03 | .09 | **-.11** | -.09 |
| Block 1. Social Support |  |  |  |  |  |  |  |  |  |  |  |  |  |  |  |  |  |  |
| 1. Affective social support | **-.17** | .03 | -.05 | **-.09** | **-** | **.29** | **.29** | **.11** | .07 | .06 | -.03 | **.18** | -.06 | -.01 | **.24** | .09 | **.25** | **.29** |
| 1. Family cohesion | **-.15** | **-.06** | **-.10** | **-.13** | **.38** | **-** | **.46** | **.17** | **.20** | **.20** | **.13** | **.34** | .08 | **.11** | **.32** | .05 | **.29** | **.39** |
| 1. Community cohesion | **-.08** | **-.14** | **-.16** | **-.19** | **.24** | **.37** | **-** | .08 | -.02 | **.13** | .02 | **.28** | **.15** | **.15** | **.28** | .00 | **.21** | **.38** |
| Block 2. Traditional practices |  |  |  |  |  |  |  |  |  |  |  |  |  |  |  |  |  |  |
| 1. Going on the land (ref=Occasionally or never) | -.03 | -.05 | -.03 | -.06 | **.10** | **.14** | **.10** | **-** | **.27** | **.24** | .05 | **.20** | .03 | -.01 | **.16** | -.03 | **.15** | .08 |
| 1. Hunting-Fishing (ref=Less than weekly) | **-.09** | .03 | -.06 | -.05 | **.13** | **.16** | .04 | **.36** | **-** | **.35** | **.12** | **.24** | **.14** | **.12** | **.15** | .02 | **.26** | .02 |
| 1. Harvesting seafood (ref=Less than monthly) | -.02 | -.04 | -.02 | -.04 | .05 | **.12** | .06 | **.25** | **.33** | **-** | **.19** | **.17** | .04 | .04 | **.22** | -.02 | .09 | .03 |
| 1. Berry picking (ref=Less than monthly) | **-.09** | -.03 | **-.08** | **-.08** | **.08** | **.15** | **.11** | **.20** | **.22** | **.15** | **-** | **.11** | **.11** | **.16** | **.15** | -.02 | .08 | **.13** |
| 1. Abilities to practice traditional activities (ref=No) | **-.12** | -.05 | -.05 | -.05 | **.27** | **.39** | **.30** | **.22** | **.22** | **.24** | **.24** | **-** | **.18** | **.11** | **.27** | -.02 | **.47** | **.39** |
| 1. Importance of spiritual values (ref=No) | -.07 | -.05 | -.03 | -.06 | **.11** | **.15** | **.12** | **.07** | **.06** | **.11** | **.08** | **.16** | **-** | **.22** | **.18** | .08 | .11 | .11 |
| Block 3. Community activities |  |  |  |  |  |  |  |  |  |  |  |  |  |  |  |  |  |  |
| 1. Participation in religious activities (ref=Less than monthly) | **-.08** | -.06 | -.02 | -.05 | .04 | **.15** | **.11** | **.09** | **.09** | **.11** | **.13** | **.20** | **.17** | **-** | **.18** | **.12** | **.15** | **.15** |
| 1. Volunteering and community activities | .00 | -.03 | .00 | -.04 | **.27** | **.27** | **.21** | **.22** | **.21** | **.19** | **.19** | **.33** | **.09** | **.23** | **-** | **.14** | **.20** | **.33** |
| 1. Healing and wellness activities (ref=No) | .06 | **.13** | .07 | **.11** | **.09** | .06 | -.03 | **.14** | **.08** | **.07** | **.13** | **.08** | .07 | .05 | **.20** | **-** | .02 | .02 |
| Block 4. Cultural Identity |  |  |  |  |  |  |  |  |  |  |  |  |  |  |  |  |  |  |
| 1. Centrality | **-.07** | .06 | .00 | -.02 | **.24** | **.25** | **.20** | **.15** | **.19** | **.17** | **.19** | **.49** | **.19** | **.11** | **.24** | **.09** | **-** | **.52** |
| 1. Connectedness | **-.11** | -.01 | -.07 | **-.09** | **.31** | **.37** | **.34** | **.13** | **.11** | **.17** | **.15** | **.50** | **.22** | **.16** | **.36** | **.13** | **.53** | **-** |

Note: Boldface indicates significant associations.

**Table S2**

*Between-block multivariate regression analyses for mental health indicators by sociocultural factors*

|  | **CES-D** | **Lifetime Ideation** | **Lifetime Attempts** |
| --- | --- | --- | --- |
|  | **Std *ß /* AORs^1^ [95% CI]** | **AORs [95% CI]** | |
|  | Women Men | Women Men | Women Men |
| **Block 1. Social support** | | | |
| Family cohesion | -0.09**/** 0.93 [0.86, 1.00] -0.18/ 0.92 [0.81, 1.04] | **- -** | 0.94 [0.87, 1.01] 0.87 [0.77, 0.98] |
| Community cohesion | - - | 0.91 [0.85, 0.97] 0.89 [0.81, 0.98] |  |
| **Block 2. Traditional practices** | | | |
| Hunting/Fishing (ref=Less than weekly) | -0.08/ 0.72 [0.54, 0.98] **-**0.18/ 0.48 [0.28, 0.82] | - - | - - |
| Ability to practice traditional activities | -0.03/ 0.99 [0.93, 1.07] 0.00/ 0.96 [0.85, 1.08] | - - | - - |
| **Block 3. Participation in community activities** | | | |
| Volunteering and community activities | - - | 0.94 [0.87, 1.02] 0.99 [0.89, 1.12] | - - |
| Healing and wellness activities (ref=No) | 0.07/ 1.47 [1.06, 2.05] 0.16/ 2.08 [1.19, 3.65] | 1.51 [1.09, 2.11] 1.61 [0.96, 2.68] | 1.51 [1.08, 2.12] 1.93 [1.11, 3.37] |
| **Block 4. Cultural identity** | | | |
| Centrality | - - | 1.17 [1.07, 1.28] 1.06 [0.95, 1.18] | 1.06 [0.98, 1.15] 1.07 [0.94, 1.21] |

*AORs,* Adjusted Odds Ratios; *CI*, confidence interval

^1^ CES-D total score was dichotomized at ≥ 9, which is also equivalent to the 66^th^ percentile.

Note: Analyses for the 12-month suicide ideation are not available due to convergence problem related to the small sample size for men. All analyses were adjusted for age, marital status, education, and employment.
